# Euthanasia

**Published:** 1897-08

**Authors:** 


### EUTHANASIA.

"When time, or soon or late, shall bring,
The dreamless sleep that lulls the dead,
Oblivion! may thy languid wing
Wave gently o'er my dying bed!"

In an editorial *The American Practitioner and News* writes:—"In dealing with the victims of chronic and incurable diseases the physician of to-day is almost as helpless as were the Asclepiadæ of the philospher's era."

The writer deplores the lamentable condition of countless people with malignant diseases never to be cured and intimates that it would be better for them if their sufferings ended in a painless, scientific manner. The Rev. Charles W. Wendt is quoted from the *New York Journal* as follows:—

"Although I strongly advocate the destruction of certain people by humane methods, I am not willing that my position in this matter should be misunderstood. All pioneers of thought have suffered misrepresentation, and I desire, as much for the benefit which may result to the world as for the defense of my own reputation as a humane man, to be thoroughly comprehended.

"To my mind one of the most deplorable and moving sights in Christendom to-day is the condition of the incurably diseased among us, especially if they are also in impoverished circumstances. Society should do all in its power to relieve this class, alleviating by all the resources known to modern medical science, and placing them above the mental distress and physical misery attaching to personal want.

"But who is to take the responsibility? Certainly not the medical man. On the field of battle some latitude may be allowable to him, but in the ordinary course of life no single individual is to be intrusted with so grave a responsibility. Shall we, then, continue as now, powerless to relieve the fearful suffering of so many unfortunates, turning a deaf ear to their entreaties and leaving them but one resource—suicide? In the name of humanity I say, No!

"A few years ago an engineer of eminence in France, afflicted with a terrible inherited disease, of which he had seen his own father perish under aggravated tortures, took his own life. In his will he explained the violence of his deed, and left to the French Government a large bequest for the establishment of a commission on euthanasia. The French Government, it was claimed, by clerical counsels, declined to accept the trust.

"Now, in order to put a check to such happenings, to put behind us all sickly, false sentimentality in the handling of cases such as I have cited, I suggest that there should be formed a properly constituted tribunal, acting only when duly called upon by suffering humanity, and then under every necessary safeguard and restraint. Let this jury consist of a number of medical men, representatives of the Government, and any others who might advantageously serve. To this tribunal have submitted any petition for examination and release which might be made by the incurably diseased and indorsed by their families. Then, if they were found incapable of recovery and sure to endure needless and great agony, the tribunal shall be empowered to gently, painlessly, and humanely put them out of suffering and give them a release into a better world.

"The least that could be urged on this terrible question is the advisability of allowing the wretch whose sufferings have driven him to suicide to have his quietus made by the authority of the State, instead of thrusting himself into the unkown with the stain of self-murder upon his soul."

It is said to have been the practice in the Dublin hospitals during the latter part of the 18th century to administer liberal doses of a fatal but not violent poison to patients suffering from incurable complaints. The custom went out and has never been revived. Among the ignorant Irish in New York there is a firm belief in a "black draught" which will be given them in Bellevue Hospital, if they are sent there for incurable disease—probably a tradition coming down from the old Dublin custom.

Among the savages inhabiting the islands of the southern Pacific, old and infirm people, who are burdens upon their family, are disposed of by being thrown from a tree top, or drowned in the sea. In this way there is a practical survival of the fittest, the weak and inefficient members of the tribe are put out of the way and only the strong and sturdy bread-winners remain. In the animal kingdom, this habit of getting rid of the useless and burdensome members of the herd or flock is well known. Preparatory to their southern flight, the storks of nothern Europe assemble and ruthlessly destroy all those who are unfit to endure the long flight to the Mediterranean and Africa.

Perhaps it would be wiser from a merely utilitarian standpoint to rid the world and society of all its useless and diseased members in order that there might be no hindrance to progress, and that the race might be strengthened.

But to put this into practice would certainly result in failure. The love for the weak and afflicted is firmly fixed in the heart of man, and no consideration for the general good would induce him to destroy the object of his pitying love. Philanthropy pales and weakens before the strong, tender affection for the diseased and afflicted.

Sentiment aside, we have no right to take human life, except in punishment for crime. Medicine is inexact. We can not prognosticate with certainty the outcome of a given diseased condition. Cure of "incurables" is a matter of every-day observation, and when we are able to assert positively that death is certain, the patient is already in extremis, and his taking off needs not to be expedited.

The love of life is strongest among those who are in danger of losing it, and no one quits it without more or less regret.

> "For who to dumb forgetfulness a prey,
> This pleasing, anxious being e'er resigned,
> Left the warm precincts of the cheerful day,
> Nor cast one longing, lingering look behind?"

Euthanasia will come when nature demands it; it is not for us to hasten it. Death is the only disease for which there is no remedy, and it is our mission to fight this last disease and not play unto its hand.

---

The medical department of the university has graduated the first woman in its history.

---

Dr. J. Mc. F. Gaston has been elected vice-president of the American Surgical Association—a most distinguished honor.

---

The University of Virginia receives $150,000 as the result of the decision of the New York court of appeals in the Fairweather will case.

---

Dr. F. S. Bourns, professor of pathology and bacteriology in the Southern Medical College, has been appointed pathologist to the Grady Hospital.

---

Dr. Floyd W. McRae has been appointed one of the surgeons to the Grady Hospital. Dr. Dunbar Roy has also been made opthalmologist to the hospital in place of Dr. Hobbs.

---

Dr. W. R. Hayden of Bedford Springs, Mass., has established a hotel and sanitarium there called the Sweetwater. We wish the doctor much success with his new undertaking.

---

Dr. Emma Wakefield, a colored woman, has been recently granted a license to practice medicine in Louisiana. North Carolina claims the precedence, having licensed a colored woman to practice in 1894.

---

A Stillwater judge has recently decided that there is no legal distinction between professional and manual services, and that under the law of Oklahoma Territory no property is exempt from execution for a doctor's bill.

